# UPR Activation and the Down–Regulation of α-Crystallin in Human High Myopia-Related Cataract Lens Epithelium

**DOI:** 10.1371/journal.pone.0137582

**Published:** 2015-09-09

**Authors:** Jing Yang, Sheng Zhou, Jianjun Gu, Minfei Guo, Honghui Xia, Yizhi Liu

**Affiliations:** 1 State Key Laboratory of Ophthalmology, Zhongshan Ophthalmic Center, Sun Yat-sen University, Guangzhou, China; 2 Department of Ophthalmology, the First People’s Hospital of Foshan, Guangdong, China; 3 Department of Ophthalmology, the Huichang County People’s Hospital, Jiangxi, China; 4 Department of Ophthalmology, Zhaoqing Gaoyao People’s Hospital, Guangdong, China; University of Colorado Denver School of Medicine, UNITED STATES

## Abstract

**Purpose:**

To investigate the expression of αA- and αB-crystallin and the unfolded protein response in the lens epithelium of patients with high myopia-related cataracts.

**Methods and Materials:**

The central portion of the human anterior lens capsule together with the adhering epithelial cells, approximately 5 mm in diameter, were harvested and processed within two hours after cataract surgery from high myopia-related (spherical equivalent ≥-10.00 diopters) and age-related cataract patients or from high myopia but non-cataractous patients (tissue were collected from ocular trauma patients with high myopia and lens trauma). Anterior lens samples from fresh cadaver normal human eyes were used as normal control (collected within 6 hours from death). Real-time PCR was performed to detect the mRNA levels of α-crystallins as well as unfolded protein response (UPR)-related GRP78, spliced-XBP1, ATF4 and ATF6. Western blot analysis was used to determine the protein level of α-crystallin, GRP78, p-IRE1α, p-eIF2α and ATF6.

**Results:**

In the lens epithelium of the high myopia-related cataract group and the age related cataract group, the mRNA and soluble protein expression of αA- and αB-crystallin were both decreased; additionally, the protein levels of ATF6, p-eIF2α and p-IRE1α and the gene expression levels of spliced XBP1, GRP78, ATF6 and ATF4 were greatly increased relative to the normal control.

**Conclusion:**

These results suggest the significant loss of soluble α-crystallin and the activation of the UPR in the lens epithelium of patients with high myopia-related cataract, which may be associated with the cataractogenesis of high myopia-related cataract.

## Introduction

High myopia is defined as an eye axial length greater than 26 mm and spherical equivalent -6.00 diopters (D) [1], and the disease affects the entire human eye [2]. A relationship between myopia and cataract has been suggested [3,4]. In high myopic patients, cataracts occur more frequently at an earlier age and progress more rapidly [5–7]. As reported by some researchers, high myopia-related cataract (HMC) is typically characterized by dark nuclei rather than other types of lens opacity [8,9].

α-crystallin, a member of the small heat shock protein family, is a protein complex composed of αA and αB subunits that is highly expressed in the lens. α-crystallin acts as a molecular chaperone and is a major lens structural protein that protects the transparent lens. In the αA-crystallin-null mice, the lenses become opaque due to the increased proliferation and decreased denucleation [10]. αB-crystallin plays a role in signal transduction, protein degradation, the stabilization of cytoskeletal structures and apoptosis [11]. Mutations in the αB-crystallin gene can lead to various pathologies, including cataracts [11]. However, research into alterations of alpha-crystallin in HMC is rare.

α-crystallin is an ATP-independent chaperone that efficiently binds to damaged or partially unfolded proteins and sequestering them to prevent widespread protein aggregation [12]. Accordingly, loss of α-crystallin in the lens results in the aggregation of unfolded or misfolded lens proteins into high molecular weight complexes, resulting in the light scattering and opacity of cataractous lenses and the loss of visual acuity. The endoplasmic reticulum (ER) is the site of membrane protein synthesis, folding, translocation and post-translation modification [13,14]. The accumulation of unfolded or misfolded proteins in the ER lumen, referred to as "ER stress"[15], activates three intracellular unfolded protein response (UPR) signaling pathways to release the protein-folding stress by increasing the ER protein folding capacity[16], reducing global protein synthesis [17], and activating ER-associated protein degradation [18–20]. UPR involves three ER transmembrane proteins activation including the PKR-like ER protein kinase (PERK), the activating transcription factor 6 (ATF6) and the inositol requiring enzyme 1 (IRE-1) [21]. GRP78 is a major endoplasmic reticulum chaperone and a master regulator of the UPR as well [22]. Activated IRE1*α* splices a 26-base intron named spliced XBP1 from an mRNA encoding X-box-binding protein 1 (XBP1). Spliced XBP1 induces the expression of genes which are involved in ER protein folding, secretion, phospholipid biosynthesis, ER expansion, and ER-associated protein degradation (ERAD) and finally activates UPR signaling [23]. Activated PERK phosphorylates eIF2α and subsequently reduces protein load in the ER by suppressing global protein translation [24]. PERK and eIF2α phosphorylation facilitates translation of transcription factor 4 (ATF4) as well [24]. ATF4 activates genes that promote restoration of normal ER function [25]. Once dissociated with GRP78, ATF6 translocates to the Golgi [26], and then is cleaved to a cleaved form [27]. The cleaved ATF6 translocates to the nucleus and up-regulates the transcription of target genes including XBP1 [28,29]. The inhibition of global protein synthesis secondary to UPR activation may enhance the lack of α-crystallin. However, the role of the UPR and the loss of α-crystallin in HMC remains unclear.

The present study examined the expression of α-crystallin and the potential activation of the unfolded protein response pathway in the lens epithelium of HMC. To rule out the high myopia or cataractous impact on UPR activation and soluble expression of α-crystallin, We set up the Non-cataractous lens with myopia group and cataractous lens without myopia group as control as well except for the normal lens control.

## Methods

### Patients

Sixty Han Chinese patients with high myopia-related dark nuclear cataracts (spherical equivalent ≥-10.00 diopters) were randomly divided into three subgroups (HMC). Sixty Han Chinese patients with age-related cataracts were randomly divided into three subgroups (ARC). Six hymopia but non-cataractous lenses (spherical equivalent ≥-10.00 diopters) were collected during traumatic lens remove surgery from ocular trauma patients within 12 hours from injury, and were divided into three subgroups (HMNC). Nine normal lenses from Han Chinese cadaver eyes (collected within 6 hours from death) served as controls in the study (Con). The age range collected for both HMC and control patients is from 40 to 50 years old. Ethical approval was obtained from the Institutional Review Board/Ethics Committee of Sun Yat-sen University (SYSU-ZOC-IRB). We certify that the study was performed in accordance with the Declaration of Helsinki. Informed consent was signed by the patients before the study was initiated. The cadaver eye tissues were obtained from the eye bank of Zhongshan Ophthalmic Center (Sun Yat-sen University, Guangzhou, Guangdong, China).

### Lens epithelium specimen collection

Human lens capsule and epithelium specimens, including the central area (5 mm in diameter) of the anterior lens capsule together with the adhering layer of epithelial cells, were obtained during cataract surgery.

### Lens epithelium sample RNA extraction and real-time PCR

Total RNA was isolated from the human lens epithelium specimens using Trizol (Invitrogen) according to manufacturer’s instructions. cDNA was generated using the Superscript First-Strand Synthesis Kit (Invitrogen). Reactions containing 1.0 μl cDNA were prepared in SYBR green master mix (Bio-Rad) and subjected to quantitative real-time PCR analysis using the Bio-Rad CFX96 Real Time System (Bio-Rad). Each reaction was repeated in triplicate, and the experiments were repeated at least three times to confirm reproducibility. Values were obtained for the threshold cycle (Ct) for each gene, and data were analyzed using the standard curve method. For each gene examined by qRT-PCR, samples were run in triplicate and the median CT value was normalized against the 18S rRNA CT. We diluted the cDNA samples of 18s group to 0.1 times of the other gene groups. The comparative CT method was used for data analysis the 18S rRNA as the reference gene to generate a log2 difference in gene expression levels. The log of average relative expression ± SEM was reported. PCR primer sequences are reported in Table 1.

**Table 1:** Gene-specific primers sequences for real-time PCR.

Primer	Forward	Reverse
αA-crystallin	5'-GAGATCCACGGAAAGCACAAC-3'	5'-GGTAGCGGCGGTGGAACT-3'
αB-crystallin	5'-CTTTGACCAGTTCTTCGGAG-3'	5'-CCTCAATCACATCTCCCAAC-3'
GRP78	5′- GACGGGCAAAGATGTCAGGA-3′	5′- GCCCGTTTGGCCTTTTCTAC-3′
s-XBP1	5′-ACACGCTTGGGAATGGACAC-3′	5′-CCATGGGAAGATGTTCTGGG-3′
ATF6	5'- CTTTTAGCCCGGGACTCTTT-3'	5'- TCAGCAAAGAGAGCAGAATCC-3'
ATF4	5'- GGGACAGATTGGATGTTGGAGA-3'	5'- ACCCAACAGGGCATCCAAGT-3'
18S	5′-TCGGCTACCACATCCAAGGAAGGCAGC-3′	5′-TTGCTGGAATTACCGCGGCTGCTGGCA-3′

### Lens epithelium protein extraction and western blot analysis

The human lens epithelium specimens were lysed in radioimmunoprecipitation (RAPI) lysis buffer supplemented with protease inhibitor cocktail, PMSF, and sodium orthovanadate (Santa Cruz Biotechnology). The lysate was sonicated and centrifuged at 13,000× g for 10 min. The supernatant was used for protein determination using the Bradford procedure (Bio-Rad) and western blot. The proteins were resolved on 12% sodium dodecyl sulfate polyacrylamide gels, transferred onto nitrocellulose membranes, and incubated with appropriate antibodies. β-actin was used as the internal control. The anti-αA-crystallin antibody (sc-22389; Santa Cruz Biotechnology, Xinhailing Company, Shenzhen, China) was used at a 1:200 dilution, the anti- αB-crystallin antibody (sc-22744; Santa Cruz Biotechnology, Xinhailing Company, Shenzhen, China) was used at a 1:200 dilution, the anti-GRP78 antibody (Abcam, Cambridge, MA) was used at a 1:2000 dilution, the anti-phospho-IRE1α antibody (Cell signaling, MA) was used at a 1:1000 dilution, the anti phospho-EIF2α antibody (Cell signaling, MA) was used at a 1:1000 dilution, and the anti-ATF6 antibody (Abcam, Cambridge, MA) was used at a 1:1000 dilution. Peroxidase-based detection was performed using chemiluminescence reagents (NEN Life Science, Xinhailing Company, Shenzhen, China). According to the manufacturer’s instructions, samples were subjected to sodium dodecyl sulfate polyacrylamide gel electrophoresis at 90 V for 30 min and then 110 V for 60 min, transferred at 0.3A for 3 hours, blocking in 5% dry milk solution for 60 min at room temperature, incubated with the primary antibody overnight at 4°C and incubated with the Horseradish peroxidase-conjugated (HRP-conjugated) secondary antibodies for 90 min at room temperature. goat-anti-rabbit IgG (Vector Laboratories) and goat anti-mouse IgG (Vector Laboratories) were used at 1:400 dilution. After incubation with secondary antibodies, membranes were developed with enhanced chemiluminescence substrate using Bio Imaging System. The bands were semi-quantified using densitometry. We repeated each experiment three times.

### Statistics

All values were expressed as the means ± SD. Statistical significance of the differences in the mean values was assessed using one way ANOVA (prism 3 software) for the three independent repeats. P-values less than 0.05 were considered to be statistically significant.

## Result

### Decreased soluble αA- and αB-crystallin expression levels in the lens epithelium of HMC patients

**Fig 1:**
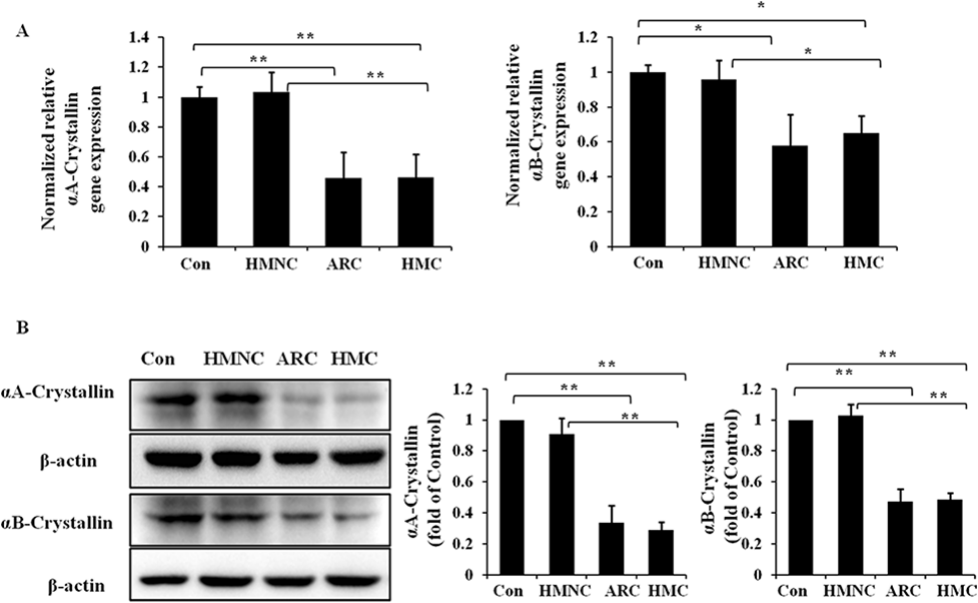
Relative αA- and αB-crystallin mRNA and soluble protein expression in the lens epithelium of HMC. RNA and soluble protein were extracted from human epithelium specimens of HMC, HMNC, ARC or normal control (Con) samples. Real-time PCR was performed to detect the mRNA levels of αA- and αB-crystallin (Fig 1A) in each group, and 18S was used as the internal control gene. Western blot assays were performed to detect the soluble αA-and αB-crystallin levels (Fig 1B), and β-actin was used as the internal control (mean ± SD, *n* = 3). *P < 0.05 and **P < 0.001.

To investigate the gene expression alteration of αA- and αB-crystallin in the lens epithelium of HMC patients, we detected the mRNA expression by real-time PCR assay. Total RNA was extracted from human lens epithelium specimens. In the current study, we found that the expressions of αA- and αB-crystallin were significantly decreased in the HMC and ARC groups compared with the normal control group (Fig 1A). However, we did not see obvious decrease of α-crystallin mRNA level in the HMNC group.

Based on the altered gene expression of αA- and αB-crystallin in high myopia-related cataract lens epithelium, we want to determine whether a similar reduction could be observed at the soluble protein level. We measured the protein levels of soluble αA- and αB-crystallin by western blot. Soluble proteins were extracted from the human lens epithelium specimens. The soluble protein levels of both αA- and αB-crystallin were significantly decreased in the HMC and ARC group but no obvious decrease in the HMNC group, when compared with the normal control. (Fig 1B).

These results indicate that the reduction of soluble αA- and αB-crystallin level might be involved in the process of HMC formation.

### Up-regulation of ER chaperone GRP78 mRNA and protein levels in HMC group

**Fig 2:**
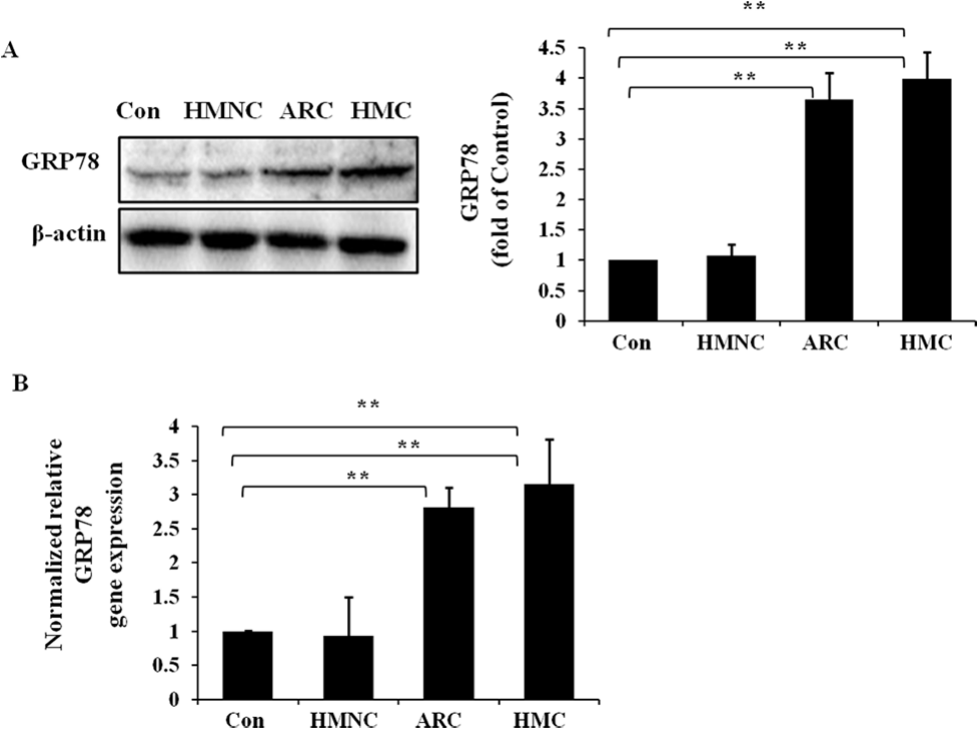
Up-regulation of ER chaperone GRP78 mRNA and protein levels in the lens epithelium of HMC patients. RNA and protein were extracted from human epithelium specimens of HMC, HMNC, ARC or normal control (Con) samples. Western blotting was performed to detect the protein expression of GRP78 (Fig 2A), and β-actin was used as the internal control. Real-time PCR was performed to detect the gene expression level of GRP78 in each group (Fig 2B), and 18S was used as the internal control gene (mean ± SD, n = 3). *P < 0.05; **P < 0.001.

To verify the activation of UPR in the lens epithelium of HMC patients, we first measured the expression of the ER chaperon GRP78 (Bip) at the protein (Fig 2A) and mRNA levels (Fig 2B). We observed that the protein level of GRP78 in the lens epithelium of HMC patients and ARC patients were up-regulated by 3.65-fold and 4.0-fold respectively relative to the normal control. Moreover, the mRNA level of GRP78 in the lens epithelium of HMC and ARC exhibited similar changes of approximately 2.81-fold and 3.15 fold relative to the normal control. However, in the HMNC group, we did not see significant up regulation of GRP78 relative to the normal control both on the RNA and protein levels. These results indicate that the GRP78 is activated in the lens epithelium of HMC and ARC patients but not in HMNC patients.

### Activation of the IRE1/XBP-1 pathway in HMC group

**Fig 3:**
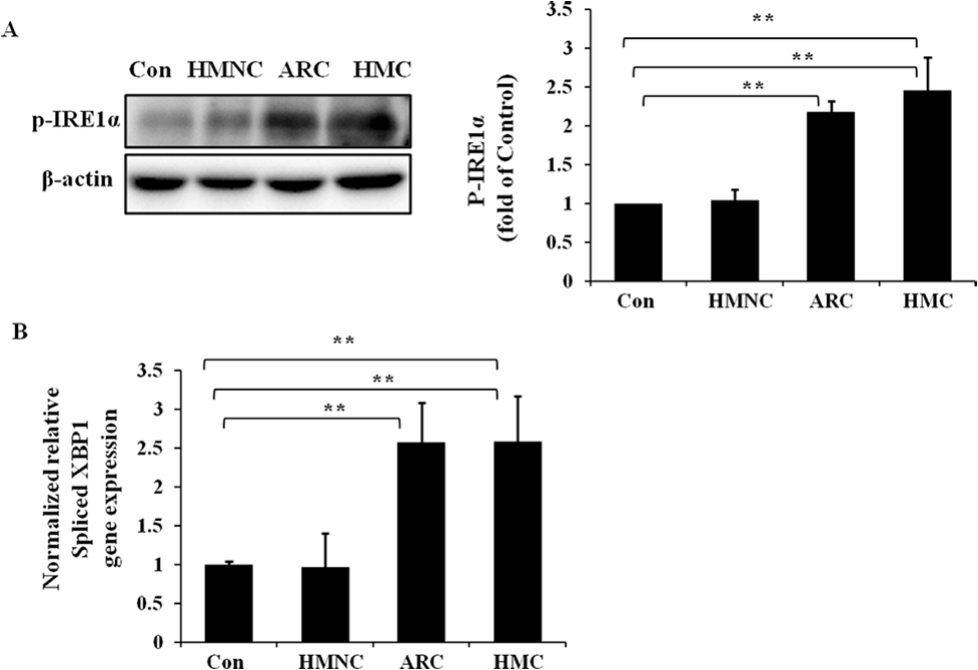
Up-regulation of p-IRE1α protein level and spliced XBP1 gene expression level in the lens epithelium of HMC patients. RNA and protein were extracted from HMC, HMNC, ARC or normal control (Con) human epithelium specimens. Western blotting was performed to detect the protein expression of p-IRE1α (Fig 3A), and β-actin was used as the internal control. Real-time PCR was performed to detect the relative gene expression levels of spliced XBP1 in each group (Fig 3B), and 18S was used as the internal control gene (mean ± SD, *n* = 3). *P < 0.05; **P < 0.001.

To investigate the activation of the unfolded protein response IRE1/XBP1 pathway in HMC lens epithelium, we measured the protein level of phosphorylated IRE1α (p-IRE1α) (Fig 3A) and the gene expression level of spliced XBP1 (Fig 3B) by western blot analysis and quantitative real-time PCR, respectively. The protein and RNA samples were extracted from human lens epithelium specimens. The p-IRE1α protein levels were significantly up-regulated by 2.45-fold relative to the normal control in the HMC group and by 2.18-fold relative to the normal control in the ARC group; the mRNA level of spliced XBP1 in the HMC group and ARC group followed a similar up-regulated trend of 2.59-fold and 2.57-fold respectively relative to the normal control. However, no significant up regulation of p-IRE1α protein level and spliced XBP1 gene expression were detected in the HMNC group.

These results suggest that the IRE1/XBP1 pathway is activated in the lens epithelium of HMC and ARC patients, but not in the HMNC patients.

### Activation of the PERK/eIF2α/ATF4 pathway in HMC group

**Fig 4:**
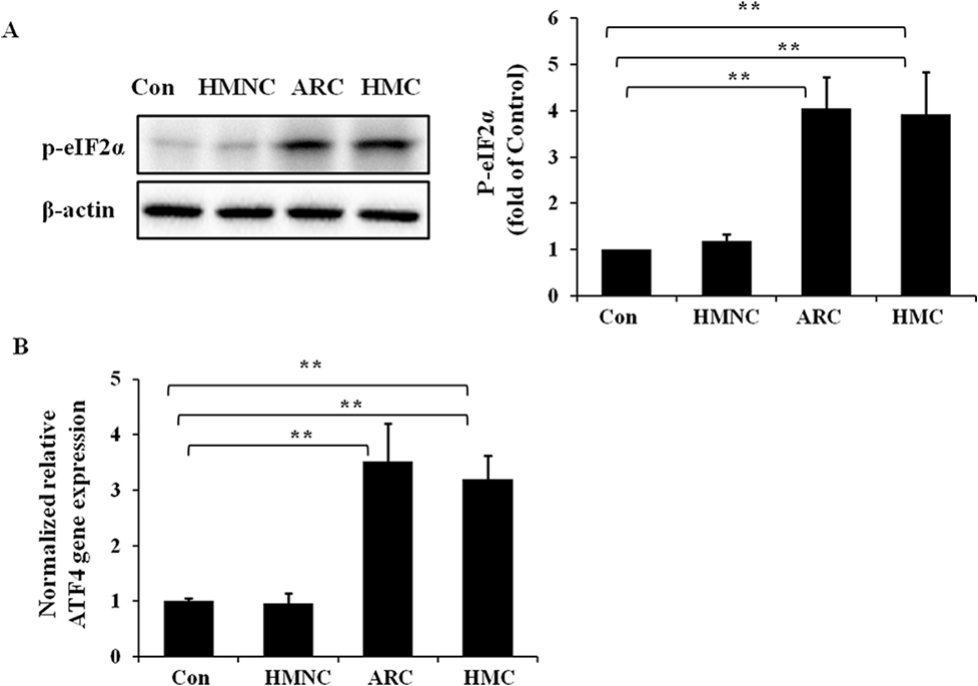
Up-regulation of p-eIF2α protein level and ATF4 gene expression level in the lens epithelium of HMC patients. RNA and protein were extracted from HMC, HMNC, ARC or normal control (Con) human epithelium specimens. Western blotting was performed to detect the protein expression of p-eIF2α (Fig 4A), and β-actin was used as the internal control. Real-time PCR was performed to detect the relative gene expression level of ATF4 in each group (Fig 4B), and 18S was used as the internal control gene (mean ± SD, *n* = 3). *P < 0.05; **P < 0.001.

To verify the activation of the PERK/eIF2α/ATF4 pathway in the lens epithelium of HMC patients, the protein level of phosphorylated eIF2α (P-eIF2α) (Fig 4A) was measured by western blot analysis, and gene expression of ATF4 was measured via quantitative real-time PCR assay (Fig 4B). The protein and RNA samples were extracted from human lens epithelium specimens. In the HMC group and ARC group, the P-eIF2α protein expression were significantly increased by 3.93-fold and 4.05-fold respectively relative to the normal control. In addition, the ATF4 gene expression in the HMC and ARC groups were increased by 3.19-fold and 3.51-fold respectively relative to the normal control. No significant up regulation of P-eIF2α protein expression and ATF4 gene expression were seen in the HMNC group.

These findings demonstrate the activation of PERK/eIF2α/ATF4 pathway in the lens epithelium of HMC and ARC patients but no activation in the HMNC patients.

### Activation of the ATF6 pathway in the HMC group

**Fig 5:**
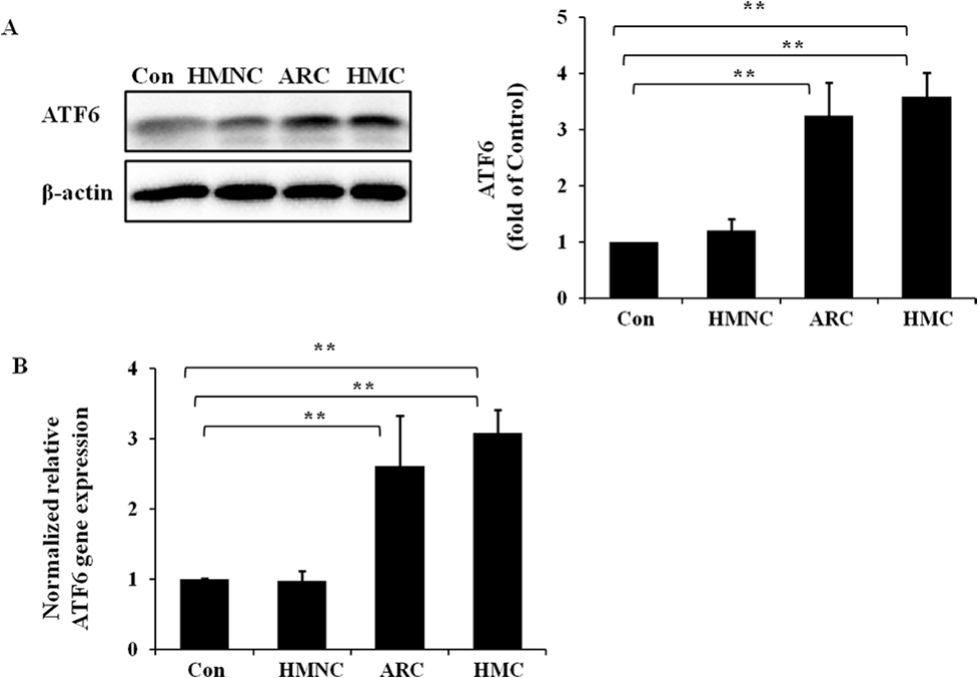
Up-regulation of cleaved ATF6 protein and gene expression levels in the lens epithelium of HMC patients. RNA and protein were extracted from HMC, HMNC, ARC or normal control (Con) human epithelium specimens. Western blotting was performed to detect the protein expression of cleaved ATF6 (Fig 5A), and β-actin was used as the internal control. Real-time PCR was performed to detect the relative gene expression level of ATF6 in each group (Fig 5B), and 18S was used as the internal control gene (mean ± SD, *n* = 3). *P < 0.05; **P < 0.001.

We sought to determine whether the ATF6 pathway was also activated in the lens epithelium of HMC patients. Western blot and quantitative real-time PCR were performed on the protein and RNA samples extracted from human lens epithelium (Fig 5). When compared with the normal control, ATF6 protein expression (Fig 5A) in the HMC group and ARC group were significantly up-regulated by 3.59-fold and 3.24-fold, respectively. The ATF6 mRNA levels (Fig 5B) in the HMC group and ARC group were significantly up-regulated by 3.09-fold and 2.61-fold, respectively.

These results demonstrate ATF6 pathway activation in the lens epithelium of HMC and ARC patients but not in the HMNC patients.

## Discussion

We describe here the reduction of soluble alpha-crystallin expression in the lens epithelium of HMC patients and its potential regulated mechanism by activation of the unfolded protein response. Lens epithelial cells are responsible for the growth and development of the entire ocular lens. In our study, reduction of αA- and αB-crystallin expression mRNA level and reduction of soluble αA- and αB-crystallin protein level were observed in the lens epithelium of HMC patients compared with the normal controls. This finding may help explain cataractogenesis. The reduction in αA- and αB-crystallin expression disturbs the normal homeostasis of the lens epithelia due to its crucial role in the survival and proliferation of these cells[30]. α-Crystallin reduction affects binding to damaged or partially unfolded proteins and subsequently affects the prevention of widespread protein aggregation[12].

When misfolded proteins accumulate within the ER, the ER chaperone glucose-regulated protein 78 (GRP78) dissociates from the UPR sensors PERK, IRE1 and ATF6 and subsequently binds to improperly folded proteins. This triggers the activation of these factors and results in the induction of three UPR-related pathways[31]. The IRE1-XBP1 pathway and the ATF6 pathway aim to produce a transcriptional response and subsequently increase the capacity of the ER; PERK pathway activation aims to induce temporary translation attenuation [18]. GRP78, also referred to as BiP, is a central regulator of ER stress due to its role as a major ER chaperone and its ability to control the activation of ER stress sensors (IRE1, PERK, and ATF6) [32]. Induction of GRP78 is a marker of ER stress and is required to alleviate ER stress and facilitate protein folding [33,34]. This role is consistent with our findings that GRP78 is significantly induced in the lens epithelium of high myopia-related cataract patients (Fig 2). This result indicates that the lens epithelium of high myopia-related cataract suffers from ER stress and that the UPR is activated.

Our results reveal the significant up-regulation of the p-IRE1α at the protein level and the up-regulation of spliced XBP1 at the gene expression level. The IRE1/XBP1 pathway is the conserved core of the UPR[18]. When the IRE1α/XBP1 branch is activated, IRE1α autophosphorylates to the p-IRE1α form and splices a 26-nucleotide intron from the mRNA encoding the UPR-specific transcriptional factor XBP1, which results in a frameshift of the XBP1 gene[35]. This frameshift then produces a more stable spliced form of XBP1 (XBP1s), which is also a potent activator of UPR genes [36]. Therefore, the induction of spliced XBP1 in our result supports the activation of the IRE1/XBP1 pathway in the lens epithelium of high myopia-related cataract. Furthermore, the up-regulation of spliced XBP1 can induce the expression of downstream genes, including genes encoding ER chaperones, such as GRP78, and protein involved in ER-associated protein degradation (ERAD) [20,37]. This regulation could be another reason for GRP78 induction in the HMC group.

Once the PERK/eIF2α/ATF4 branch of the UPR is activated, PERK undergoes autophosphorylation to the p-PERK form. Subsequently, p-PERK phosphorylates the α-subunit of eIF2 to form p-eIF2α[17], which results in the global arrest of protein synthesis due to reduced translation[18]. In addition, p-eIF2α can induce another transcription activator, ATF4[38]. ATF4 then induces a subset of UPR genes, including XBP1. In our study, the protein expression of p-eIF2α and the mRNA level of ATF4 in the lens epithelium of HMC were dramatically increased, which reflects the activation of the PERK/eIF2α/ATF4 pathway. The global arrest of protein synthesis downstream of activation of the PERK/eIF2α/ATF4 branch may lead to the reduction of α-crystallin in HMC group, as we described in Fig 1.

ATF6 is also an ER stress sensor. Upon ER stress, ATF6 is transported from the ER to the Golgi and cleaved to an N-terminal 50-kDa protein (p50ATF6) [27]. The cleaved ATF6 then moves to the nucleus to promote the expression of a subset UPR target genes [28,39]. In our study, the induction of cleaved ATF6 protein and ATF6 gene expression suggests ATF6 branch activation in the HMC group.

Nevertheless, our results revealed that GRP78, p-IRE1α, spliced XBP1, p-eIF2α, ATF4 and ATF6 were all significantly increased in the ARC group as well, which suggest UPR activation in cataractous but non-high myopic lenses also. In addition, we did not get evidence that UPR related markers were activated in high myopic but non-cataractous lenses. In our findings, the high myopic impacts could be ruled out of the UPR activation and soluble α-crystallin reduction, however, the cataractous impact such as cytotoxicity caused by fiber cell necrosis during cataractogenesis cannot be ruled out.

In summary, these results demonstrate the reduced expression of the molecular chaperones αA- and αB-crystallin and the activation of three UPR pathways in the lens epithelium of high myopia-related cataract. As discussed above, the reduced expression of α-crystallin might trigger the UPR, and the activation of UPR could result in decreased synthesis of α-crystallin. These findings provide us a new explanation for cataractogenesis mechanisms in high myopia-related cataracts. However, it remains unclear which is the triggering event or whether these changes occur independently with crosstalk between α-crystallin expression and UPR activation or both of these changes are all induced by cytotoxicity caused by fiber cell necrosis during cataractogenesis. Further examination of the relationship between α-crystallin expression and UPR activation should be the focus of future studies.
